# Why do women invest in pre-pregnancy health and care? A qualitative investigation with women attending maternity services

**DOI:** 10.1186/s12884-015-0672-3

**Published:** 2015-10-02

**Authors:** Geraldine Barrett, Jill Shawe, Beth Howden, Dilisha Patel, Obiamaka Ojukwu, Pranav Pandya, Judith Stephenson

**Affiliations:** Department of Clinical Sciences, Brunel University London, Uxbridge, UB8 3PH UK; School of Health Sciences, University of Surrey, Guildford, Surrey GU2 7XH UK; Reproductive Health, Institute for Women’s Health, UCL, London, WC1E 6AU UK; University College London Hospitals, 235 Euston Road, London, NW1 2BU UK

**Keywords:** Preconception care, Pre-pregnancy, Pregnancy, Folic acid, Micronutrient supplementation, Qualitative

## Abstract

**Background:**

Despite the importance attributed to good pre-pregnancy care and its potential to improve pregnancy and child health outcomes, relatively little is known about why women invest in pre-pregnancy health and care. We sought to gain insight into why women invested in pre-pregnancy health and care.

**Methods:**

We carried out 20 qualitative in-depth interviews with pregnant or recently pregnant women who were drawn from a survey of antenatal clinic attendees in London, UK. Interviewees were purposively sampled to include high and low investors in pre-pregnancy health and care, with variation in age, partnership status, ethnicity and pre-existing medical conditions. Data analysis was conducted using the Framework method.

**Results:**

We identified three groups in relation to pre-pregnancy health and care: 1) The “prepared” group, who had high levels of pregnancy planning and mostly positive attitudes to micronutrient supplementation outside of pregnancy, carried out pre-pregnancy activities such as taking folic acid and making changes to diet and lifestyle. 2) The “poor knowledge” group, who also had high levels of pregnancy planning, did not carry out pre-pregnancy activities and described themselves as having poor knowledge. Elsewhere in their interviews they expressed a strong dislike of micronutrient supplementation. 3) The “absent pre-pregnancy period” group, had the lowest levels of pregnancy planning and also expressed anti-supplement views. Even discussing the pre-pregnancy period with this group was difficult as responses to questions quickly shifted to focus on pregnancy itself. Knowledge of folic acid was poor in all groups.

**Conclusion:**

Different pre-pregnancy care approaches are likely to be needed for each of the groups. Among the “prepared” group, who were proactive and receptive to health messages, greater availability of information and better response from health professionals could improve the range of pre-pregnancy activities carried out. Among the “poor knowledge” group, better response from health professionals might yield greater uptake of pre-pregnancy information. A different, general health strategy might be more appropriate for the “absent pre-pregnancy period” group. The fact that general attitudes to micronutrient supplementation were closely related to whether or not women invested in pre-pregnancy health and care was an unanticipated finding and warrants further investigation.

## Background

The recognition of the importance of women’s health before pregnancy and their health behaviours around the time of conception has led to a growing interest in pre-pregnancy care over the last decade [1–4]. Evidence suggests that improvements in women’s health and health behaviours have the potential to improve pregnancy outcomes and the health of the child beyond birth [5–9].

Although specific guidelines may vary, there is a broad consensus that pre-pregnancy care includes folic acid supplementation to prevent neural tube defects, stopping smoking, stopping or reducing alcohol consumption, achievement/maintenance of a healthy weight, screening for infection, avoidance of occupational and environmental hazards, and for women with pre-existing conditions (e.g. diabetes, epilepsy, hypertension, hypothyroidism, asthma and major mental health disorders) additional care and medication review [1, 2, 10, 11]. In the UK, public health information on the need to take folic acid prior to pregnancy has been in place for over 20 years [12, 13].

Despite the importance attributed to good pre-pregnancy care and its potential to improve pregnancy and child health outcomes, relatively little is known about women’s behaviour in relation to pre-pregnancy health and care. One of the most investigated areas has been the use of folic acid by women before pregnancy, which has consistently shown that sizeable proportions of women do not take it before pregnancy [14–19]. Other health behaviours in the pre-pregnancy period have been less investigated although the available evidence suggests that only a small proportion of women follow recommendations relating to lifestyle and diet [18, 20–23].

In order to gain a broad picture of how women prepare for pregnancy, our research team surveyed nearly 1200 women attending maternity services in London, UK. The findings of this survey which have been reported elsewhere [24] showed that: 51 % of women (and 63 % of those with a planned pregnancy) took folic acid supplements before pregnancy; 31 % of women (and 38 % of those with a planned pregnancy) changed their diet before pregnancy; 21 % of women reported smoking and 61 % reported drinking alcohol in the 3 months before pregnancy, with 48 % of smokers and 41 % of drinkers reducing or stopping before pregnancy. Relatively few women received advice from health professionals prior to pregnancy on a healthy diet, healthy weight, caffeine, alcohol or smoking. In the sample overall, 25 % had a medical condition potentially requiring specialist pre-pregnancy advice, 26 % were taking regular medication, and 27 % had pregnancies classified as unplanned or ambivalent. In order to gain insight into *why* women invested in pre-pregnancy health and care, we carried out qualitative interviews with a sub-sample of the survey. We report the findings of those interviews in this paper, focusing on women’s explanations, feelings and attitudes towards pre-pregnancy health and care.

## Methods

In order to investigate why women do, or do not, invest in pre-pregnancy health and care we chose qualitative methodology, using in-depth interviews, to allow us the flexibility to probe personal accounts and reasoning and for the exploration of interviewees’ opinions and ideas. Within a framework of generic qualitative research [25], our methodological approach can be broadly described as qualitative description [26], with a subtle realist stance [27]. By this we mean that we believe there are “independent, knowable phenomena” [27] that can be gained from women’s accounts, whilst acknowledging that these accounts are socially constructed and a product of the particular interview/study encounter [27, 28].

The interviewees were chosen from a sample of 374 women who had consented to be contacted for interview after taking part in a survey of 1173 antenatal clinic attendees at three London (UK) hospitals [24]. From the sample of consenters, a purposive sample was drawn. A pragmatic, *a priori* decision was made to recruit approximately half high investors in pre-pregnancy care and half low investors (operationalised as those who reported carrying out six or more pre-pregnancy activities in the survey versus those who carried out one activity or no activity). Within the low and high investors, maximum variation sampling in relation to socio-demographic characteristics was carried out (operationalised by aiming for at least one woman in each group who was younger (<=23), older (= > 37), not White British, not living with a partner/husband, and who had a pre-existing health condition. We recruited women to the interview sample until we had 20 interviewees (we attempted to contact 29 women in this process, with the contact information for five women no longer valid, three women declining an interview because of lack of time or interest, and one woman agreeing to an interview and then not being available for interview). The sample size of 20 interviewees was a pragmatic choice based on a mixture of factors [29], including: the scope of the study, the research funding available to us, and our previous experience of interview samples in qualitative research.

All interviewees gave signed, informed consent for the interviews and received a £10 shopping voucher as a thank you afterwards.

The 20 interviews were carried out by five interviewers (BH-8, GB-5, DP-5, JSh-1, OO-1). All interviewers were part of the research team, most were experienced qualitative interviewers/researchers, and no member of the team had responsibility or involvement in the clinical care of the interviewees. Nine of the interviews were carried out face-to-face and 11 by telephone according to the preference of the interviewee. Interviews tended to last 45–60 min.

In the interviews, women were asked about their socio-demographic background, the circumstances of conception (i.e. their intentions, feelings, personal situation), and about their actions (if any) to prepare for pregnancy. The topic guide contained a list of likely pre-pregnancy actions which interviewers could use as a prompt if needed (the list included: supplements – folic acid, multivitamins for pregnancy, ordinary multivitamins, vitamin D, iron, omega 3, vitamin C, zinc; lifestyle actions – eating a healthy diet, weight, caffeine, alcohol, smoking, drugs, including street drugs, prescription drugs, over-the-counter drugs; and health care advice – seeing a health professional for advice about getting pregnant, immunizations, dental checks, sexually transmitted infections, and stopping contraception). Throughout the discussions of actions, the interviewers probed the reasons for actions and, as far as possible, the reasons for no action. The next topic sought information on who women discussed pre-pregnancy health and care with. Then towards the end of the interview, as a mechanism to stimulate discussion, we asked women to consider the issue of the large proportion of unplanned pregnancies each year in the UK and a scenario whereby women of reproductive age carry out pre-pregnancy activities, such as taking folic acid routinely, in case of pregnancy. We asked women how they would feel personally about doing this. Finally, we closed the interview by asking interviewees how they thought information about pre-pregnancy health and care could be given to women.

The interviews were digitally recorded and transcribed verbatim by a reputable transcription company. Thematic analysis was carried out using the Framework method [30]. The Framework method has five stages: 1) familiarisation; 2) identifying a thematic framework; 3) indexing; 4) charting; and 5) mapping and interpretation. The first three stages of the analysis were managed using NVivo 9.0 software and charting was recorded on an Excel spreadsheet. Mapping and interpretation was an iterative process, involving note-making and writing, with continual movement between the chart, codes, and full transcripts. The Framework method ensures a rigorous, grounded analysis by helping researchers stay close to the data whilst developing their description of phenomena and their concepts. The analysis was led by GB (a sociologist by training and health researcher), with JSh (a nursing professional and qualitative researcher) independently assessing coding decisions and charting. All interviewers and authors contributed to the report.

The qualitative study was approved by the National Research Ethics Service, NRES Committee London – Bromley (REC reference 11/LO/0881). The approval was given as part of a larger study of preconception health and care in England.

## Results

### Sample achieved

Of the 20 interviewees, six were in their 20s (with the youngest aged 23), six aged 30–34, six aged 35–39, and two women were aged 40. Eight women were living with their husbands, nine with a male partner, and three without a partner/husband (two of whom had a non-resident partner). Fourteen interviewees had degree level (or above) education. Eleven of the interviewees were white British women, a further four were born in Britain and classified themselves as non-White (Black African, Arab, Indian and Pakistani), three were white women born in, respectively, Australia, USA and Ireland. One woman was born and raised in Africa and another was born in Iran but came to the UK as a young child. Eight women reported health conditions that were active just before pregnancy (two with polycystic ovarian syndrome, and one each with: sickle cell anaemia, ulcerative colitis, asthma, eczema and food allergies, acne, and irritable bowel syndrome). A further woman reported having breast cancer in the past, and two reported previously having had depression (one with no other health condition, one with). Two women were still pregnant at the time of their interview; all the others had babies under six months old. Thirteen of the interviewees were, or were about to be, first time mothers, seven were mothers already (six having their second child and one her fourth). Interviewees’ characteristics according to the *a priori* sampling criteria and their recruitment group are shown in Table 1.

**Table 1:** Interviewees’ characteristics by group according to the a priori sampling criteria

	Survey		Qualitative groupings		
Sampling criteria	Low investors (11)	High investors (9)	Absent pre-pregnancy period (6)	Poor knowledge (5)	Prepared (9)
Young age < =23	1	0	0	1	0
Older age = > 37	2	3	1	1	3
Not ‘White British’	4	5	3	1	5
Not living with husband/partner	2	2	2	1	1
Pre-existing health condition	4	4	3	1	4

### Experience of pre-pregnancy health and care in this pregnancy

We classified interviewees into three broad groups regarding their experience of pre-pregnancy health and care in this pregnancy: the “prepared” group, the “poor knowledge” group, and the “absent pre-pregnancy period” group. Broadly, the prepared group corresponded with the “high investors” selected from the survey and the other two groups with the “low investors”, with the exception of two women whose interview accounts differed slightly from their survey responses. Interviewees’ characteristics according to the a priori recruitment criteria and their assigned group are shown in Table 1.

#### The “prepared” group

The “prepared” group comprised nine women who had highly planned pregnancies, including one IVF pregnancy. Two of the women were in their early 30s and the rest were 35 or over. Six women were married, three were living with partners, five were first time mothers, and four were mothers already (having had their second child). Four women were white British women, and all had at least degree level education.

The defining feature of this group is that they had all made a conscious decision to become pregnant and began pre-pregnancy activities which they continued, by and large, consistently into pregnancy. All took folic acid before pregnancy and additionally, most commonly, took a pregnancy multivitamin or additional vitamins (six women), stopped or reduced alcohol (five women) and ate more healthily (three women). The women in this group described their pregnancy preparations in matter-of-fact way, and most began additional activities once the pregnancy was confirmed, e.g.:

“Well I’d been trying for about 6 months, so um … by the time you know I did get pregnant I was a bit fed up really. You know you take those vitamins and things, which basically I already knew, you know, from my knowledge that, you know, you’d have to take folic acid and … yeah, various vitamins beforehand to help you, you know, conceive and also to ensure the health of your baby. I wasn’t doing anything that much different. I was exercising and working … that’s about it really” (Interview 3, age 35, prepared group).

“I cut down on all the life style-y stuff like smoking and drinking and I was trying to exercise a bit more, but not … I wouldn’t say I was doing it extremely. Like I wasn’t … I didn’t completely stop my life or completely not go out or anything like that so it wasn’t … um, yeah, I did a little bit. But, obviously, when I fell pregnant, then it was just … it was so easy, it was easy to stop 'cause you knew that there was something else. […] I was on folic acid before that [pregnancy], I was taking it regularly” (Interview 7, age 36, prepared group).

#### The “poor knowledge” group

The “poor knowledge” group comprised five women. The women’s ages ranged from 23 to 40 (two in their twenties, two in their early thirties, and one 40 year old). Two were married, two lived with partners and one had a non-resident partner. Four were first time mothers. Four were white British women, and three had at least degree level education.

The pregnancies of the women in this group were either highly planned (three women) or mostly planned (two women), however their pre-pregnancy activities (or lack of them) coincided with their reported lack of knowledge about pre-pregnancy health and care. One woman cut down on international travel in her preparations to get pregnant, one downloaded a pregnancy app onto her iPad to help her know when her fertile period was, and one saw her consultant to ask advice about her ulcerative colitis medication in relation to pregnancy, however all three reported that they did not become aware of any other pregnancy related information. The last woman also had an x-ray in the period between getting pregnant and having the pregnancy confirmed. None of the five took folic acid prior to pregnancy, nor did they know that it should be taken before pregnancy. None of them carried out any other pre-pregnancy activities. One woman, reflecting at the end of the interview, described the situation:

“I don’t know, you just assume that you just get pregnant and then you go to your GP and everything falls in place from there. I didn’t know that there were things that you should be doing prior to becoming pregnant. You don’t really plan it … although I was planning it” (Interview 20, age 30, poor knowledge group).

#### The “absent pre-pregnancy period” group

The “absent pre-pregnancy period” group comprised six women. Four of the women were in their twenties and two in their 30s. Four women were living with partners, one had a non-resident partner, and one had no partner. Four were first time mothers. Three were White British women, and two of the women had at least degree level education.

All six women described their pregnancies as unplanned, however with three of the women there were also inconsistencies and suggestions of ambivalence or varying levels of intention in their accounts. The striking feature of these women’s accounts was the lack of discussion about pre-pregnancy health and care. As the women considered, or presented, the conception as outside their control (e.g. “it was a complete surprise”, “it just happened”) therefore they did not consider themselves as having had responsibility in terms of pre-pregnancy preparation. Even trying to discuss ideas about, and attitudes towards, pre-pregnancy health and care was difficult as the women quickly moved to considering the questions in light of pregnancy, not pre-pregnancy, e.g.:

Interviewer: “Do you think there is enough information out there for women that are planning pregnancies?”

R: “Um … so I mean you know I was older when I had the baby. I read a lot, I’m… you know I’m well educated. And as soon as I found out I was pregnant I was relatively proactive in finding out as much as I could about what I could do to kind of maximise the baby’s wellbeing and so on. I think if I was younger … if I’d lived a bit less and if I didn’t know as much as I know about to kind of find information, I don’t know if I’d find it all that easy to access, do you know what I mean?” (Interview 13, age 39, absent pre-pregnancy period)

Four of the five women did not take folic acid before pregnancy and knew relatively little about it. The following comment typified descriptions in this group:

“… because we weren’t especially kind of organised about getting pregnant I didn’t do anything until I found out I was pregnant, and then it was probably at the point when I spoke to my GP that I started to take folic acid. I wasn’t especially well educated about … I knew I should take it but I didn’t know exactly how quickly I should start to take it when I got pregnant if you know what I mean” (Interview 13, age 39, absent pre-pregnancy period group).

The one woman who was taking folic acid before pregnancy was taking it for her sickle cell anaemia, not in preparation for pregnancy. No other pre-pregnancy activity was carried out.

### Consulting health professionals about pregnancy

Five women in the sample discussed with doctors, in advance of pregnancy, the fact they were planning to get pregnant (four from the prepared group and one from the poor knowledge group). Two women, both from the prepared group, consulted their General Practitioners. The first of these women saw her GP for IUD removal; the only advice she remembered being given in the consultation was to think about contraception after pregnancy. The second of these women consulted her GP specifically to ask for pre-pregnancy care advice:

“So I said [to GP] ‘I’m 35 this year and my partner and I would like to start having kids, what should I do?’ because I wondered with the folic acid and … you know, what sort of lifestyle changes I should have and blah, blah, blah. And she said the best thing to do is to enjoy being together, she said one of the biggest problems with falling pregnant with young couples is that they put so much pressure on themselves, it then causes problems with being able to fall pregnant […] I’d read something about folic acid and I said ‘What do you think, folic acid?’ she was like ‘Yeah, yeah, take folic acid’ but I think I was kind of instigating that" (Interview 7, age 36, prepared group).

This interviewee was also told by her GP that the average time to get pregnant was 18 months (which is inaccurate). It is worth noting, however, that she was very happy with the advice she received from her GP, feeling that the GP was reassuring and supportive. The next three women (two from the prepared group, one from the poor knowledge group) discussed with hospital doctors their plans to get pregnant. The first woman discussed her plans with the gynaecologist who was treating her for polycystic ovary syndrome; as part of their discussions the doctor gave her advice about folic acid, which she knew anyway. One woman saw her dermatologist for advice about the safety in pregnancy of her acne medication (she was advised to stop); the consultation focused only on this topic and she received all her nutritional and supplement advice from her sister, a naturopathic nutritionist. The third woman (from the poor knowledge group, discussed earlier) saw her consultant to ask advice about her ulcerative colitis medication in relation to pregnancy. The consultation focussed only on the medication and she did not receive any other advice. In this case the woman had poor knowledge of folic acid and nutritional supplementation and consequently did not begin folic acid supplementation before pregnancy.

One further woman, from the prepared group, saw a nutritionist about supplementation for pregnancy. Another woman, from the prepared group, described how there had been a promotion of pre-natal multivitamins at her GP surgery, with information and free trial bottles available. She started taking these when she was not pregnant and switched to another brand when she found out she was pregnant. She was already aware of the need to take folic acid and was happy to take the opportunity of a free sample.

### Advice from family and friends

In general, family and friends were a poor source of advice on pre-pregnancy health and care. Even in the few cases where interviewees told family or friends that they were trying to get pregnant, they reported being given little or no advice. In contrast, advice from family and friends was much more forthcoming once a pregnancy was announced. Two women (one from the poor knowledge group and one from the absent pre-pregnancy period group) reported that they were given advice about folic acid in pregnancy by family after they had announced they were pregnant.

Women from the prepared group were most likely to say that they had a knowledgeable set of friends, who knew about folic acid and pre-pregnancy activities, but even this was not so for all women in the group. Also, two women from the prepared group said that they themselves would be cautious about giving advice to others, for fear of appearing to pry, e.g.:

“Just the usual folic acid stuff that they know, and then like … doesn’t really discuss these things [inaudible] to discuss what they are and aren’t doing to try and get pregnant. Especially when it’s taking a bit of time as well [inaudible] they don’t want to … don’t really want to discuss it, touchy subject, so I don’t really pry too much into it” (Interview 3, age 35, prepared group).

### Knowledge about folic acid

At the time of interview, knowledge that folic acid was necessary *in pregnancy* was high among interviewees. Women reported being told about folic acid by health professionals when they were pregnant, and by family and friends, and reported that they felt comfortable talking to pregnant family and friends about folic acid.

What was very poorly understood was that folic acid needed to be started *before* pregnancy, and was required right at the beginning of pregnancy. Even among women in the prepared group, who all started folic acid before pregnancy, knowledge as to why this was necessary was very poor. The following comments were typical:

“…like when they said just take it [folic acid] like for the first few months… didn’t know like before it would help as well” (Interview 5, age 23, poor knowledge group).

R: “Oh wow, three months before?!”

Interviewer: “Yeah”

R: “Oh, I didn’t know that”.

Interviewer: “But you were doing it.”

R: “I was doing it anyway, thank God, I was taking it every day. Oh wow!” (Interview 11, age 36, prepared group)

“…I’ve always known about folic acid in general. I didn’t know you should start taking it like even when you’re trying to get pregnant. […] Then it made sense to me once I learned a little bit about it.” (Interview 18, age 34, prepared group)

Knowledge about action of folic acid was often vague, e.g.:

“..that helps their bones form and stuff, it’s better to take it ‘cause something can happen .. but I can’t remember what they said” (Interview 5, age 23, poor knowledge group).

“…it’s something to do with the baby needing something for development, isn’t it?” (Interview 7, age 36, prepared group).

“I know it’s to stop abnormal… I don’t really … I think it’s to stop abnormalities of the fetus, in the development of the fetus, but I’m not quite sure” (Interview 19, age 37, prepared group).

Only two women in the whole sample, both in the prepared group, mentioned the prevention of spina bifida in relation to folic acid.

Apart from those who found out about folic acid in this pregnancy, most women could not recall when they had started to acquire the knowledge about folic acid nor where from, often identifying multiple sources.

### Knowledge about other pre-pregnancy care activities

Outside of folic acid, women knew about a range of pre-pregnancy activities (most frequently stopping smoking, stopping/reducing alcohol, eating a healthy diet), however quite often the information was not to be at the forefront of women’s minds and was often only discussed after prompting. Below are two examples, the first when the interviewer is probing knowledge:

Interviewer: “Could I run through just a short list of things and you can tell me whether anyone ever mentioned these things to you in terms of things to consider before you get pregnant.”

R: “Yeah.”

Interviewer: “Is that okay? So you’ve said about folic acid … multivitamins for women trying to conceive.”

R: “No.”

Interviewer: “Right. How about vitamin D?”

R: “No.”

Interviewer: “Iron.”

R: “No.”

Interviewer: “Omega 3.”

R: “No.”

Interviewer: “Vitamin C or zinc.”

R: “No.”

Interviewer: “Right. How about things like eating a healthy diet and being the right weight for your height?”

R: “No. Well no one spoke to me about it, but that I guess is something I knew.”

Interviewer: “Yeah. You mentioned that you used to drink but you’d stopped drinking before you got pregnant. Why did you stop drinking?”

R: “Not before, no … just as I knew, when I knew I got pregnant, that’s what I meant yeah.”

Interviewer: “Ah right, okay. And you’ve never smoked have you?”

R: “Sorry, what?”

Interviewer: “You’ve never smoked have you?”

R: “No, no, I don’t smoke.”

Interviewer: “Right, okay. Are you aware that sometimes women will check out whether their immunisations are up-to-date before they get pregnant?”

R: “No.”

Interviewer: “Okay. And how about dental checks or sexual health checks?”

R: “Yes, I knew that some people do sexual health checks, not dental.” (Interview 1, age 30, poor knowledge group).

The second example is interesting in the same respect. The interview is with a woman (from the prepared group) who is a dentist. After asking about knowledge, the interviewer uses the opportunity to ask the woman about her professional advice on pre-pregnancy care. Initially, the interviewee has to think about her answer but then becomes fluent:

Interviewer: “What about things like, say, your immunisations and … well your dental checks and things like that, is that something that you looked into during that time, when you were trying to get pregnant?"

R: “Dental checks, I already know that my teeth are fine. And immunisations, I know like I’m up to date with all my immunisations. So … don’t really need to like do anything further.”

Interviewer: “So you’re a dentist, right. Do women ever ask you advice say on their … if they’re trying to get pregnant or if they are pregnant, do women ever ask you for advice as a dentist?”

R: “Not really no.” [laughs]

Interviewer: “If they were, do you know what kind of things that you would need to tell them or want to advise them on?”

Respondent: “Um … I suppose you could just say you know eat healthy. Obviously avoid smoking, alcohol, you know maintain your weight at a reasonable level, steady level, within the limits of what is normal … normal BMI. And um … yeah just take your supplements.”

Interviewer: “What about from an actual dental perspective?”

R: “Well dental perspective um … keep your … you know brush your teeth twice a day, make sure you floss twice a day if you can, keep your teeth clean, go to see the dentist regularly, use a fluoridated toothpaste, and you know … that’s it really. Just make sure you haven’t got any residual problems in your teeth because it can have an effect on your health. Especially you know if you haven’t been to the dentist for a long time and they haven’t taken x-rays and then you know you have a problem during pregnancy, it’s very … you know we can’t really take x-rays then, we try and avoid that unless it’s an absolute emergency, so then it’s better if you like see the dentist, you’ve seen a dentist beforehand, before you even start trying for family so that you know we’ve got records, proper records for you. […] You know nobody wants to have an x-ray taken when they’re pregnant, and then you know it’s very difficult because you can only … you can do very limited things. And if we need to do an extraction we need to have an x-ray, that’s a legal requirement. So then you know it’s very tricky then” (Interview 3, age 35, prepared group).

### Being prepared for pregnancy

When we asked women to consider the issue of the large proportion of unplanned pregnancies each year in the UK and a scenario whereby women of reproductive age carried out pre-pregnancy activities, such as taking folic acid routinely, in case of pregnancy, we asked women to tell us how they would feel personally about doing this. It was striking that the primary response of four women in the sample (two from the prepared group and two from the absent pre-pregnancy period group) was strongly hostile. They felt it was patronising to women (assuming that they could not control their fertility) and that it was the State dictating personal behaviour, e.g.:

“I don’t like that at all, I don’t like the idea that we’re treated as kind of breeders first and foremost. […] I’d feel very offended at the idea that from the age of kind of 15 I should be taking folic acid in case I get knocked up – no thanks. Um, no I wouldn’t do that" (Interview 13, age 39, absent pre-pregnancy period group).

A more common, and less emotional, response from women was that simply they would not do this as they would not feel it necessary, e.g.:

“But if you tell me to take something like folic acid for the sake of it and I’m not trying to get pregnant, I won’t … why would I do that? On the off-chance?" (Interview 12, age 40, poor knowledge group).

“In practice would I have done it? I’m not sure, it would have depended on you know … I think I can be pretty slapdash at remembering to do … say taking a tablet anyway. And it would depend on the cost […] you know I wouldn’t have taken them just on the off-chance I guess. […] Like say three or four years ago … whereas in a situation where I didn’t want to be pregnant anyway … then I’d rather invest my energy into contraception than preparing for a possible pregnancy if you know what I mean” (Interview 9, age 29, poor knowledge group).

This type of response came from across the groups (two women from the prepared group, three from the poor knowledge group, and two from the absent pre-pregnancy period group). Interestingly, in a number of interviews it was at this point that anti-supplement feelings began to be expressed for the first time. A minority of women found it difficult to consider this question at all, reinterpreting it in ways that changed the meaning. Only two women, both from the prepared group, gave a positive response to this question, saying that they would be happy to take folic acid all the time, although one said she wanted it incorporated into her multivitamin rather than have to take a separate pill. Women who had described themselves as having an unplanned pregnancy did not perceive any greater relevance of this question.

### Ways of promoting pre-pregnancy health and care

We asked women what they thought the best ways were for promoting pre-pregnancy health and care. Women gave a variety of answers including: advertising on television, trains, buses; online information, including via social networking sites; leaflets in GP surgeries. Most women, when questioned, said that they would not mind receiving information about pre-pregnancy health and care at family planning appointments, at smear test appointments, or from GPs or other health professionals proactively. There were, however, comments at this point in the discussions that women would probably ignore this information unless they were interested in getting pregnant, e.g.:

“It’s a hard one isn’t it, 'cause I think unless someone’s planning a kid they don’t really care do they? Again I just don’t know. I think if it was a national campaign it would be easy to do posters and things, but then at the same time would people take any notice?” (Interview 6, age 27, absent pre-pregnancy period group).

“I think I’d sort of choose to ignore it if it wasn’t something I was thinking about” (Interview 19, age 37, prepared group).

Three women (two from the prepared group, one from the absent pre-pregnancy period group) suggested that the non-pregnancy benefit of supplements need to be stressed if women were to be encouraged to take them.

### Attitudes to micronutrient supplementation

Women’s attitudes to micronutrient supplementation (i.e. vitamins, minerals, essential fatty acids, etc.) emerged during the interview discussions. Seven of the nine women from the prepared group expressed positive attitudes to supplementation, all taking multivitamins or separate supplements outside of pregnancy as well as during pregnancy. The women described these actions as positive for their health and, during pregnancy, the health of their baby. Two of the women in this group had slightly different views. One, who was very knowledgeable about micronutrients and her diet, used supplements outside of pregnancy and during pregnancy, and only expressed ‘anti-supplement’ views when considering in the interview the hypothetical scenario of women of reproductive age carrying out pre-pregnancy activities in case of pregnancy. The other, who was also knowledgeable about diet, strongly believed that sufficient micronutrients could be gained from diet alone, however, she accepted the recommended advice about folic acid and was happy to take this before and during pregnancy.

In contrast to the prepared group, anti-supplement views and/or a dislike of taking pills was the predominant view expressed by the poor knowledge group. Similarly, with the absent pre-pregnancy period group, a couple of women expressed strong anti-supplement views (i.e. that the supplements were not necessary) and a couple just reported themselves as not being very good at taking pills. There appeared to be no history of micronutrient supplementation outside of pregnancy in either of these groups.

When women expressed anti-supplement views they tended to locate these opinions within a theme of suspicion or cynicism about the necessity of supplements and sometimes with overtones of the ‘natural as good, unnatural as bad’ narrative, e.g.:

“I’m not really a fan of vitamins. I just feel that they’re a bit of a ploy to make people spend money unnecessarily, and I think I get … I eat a very healthy, sort of balanced, healthy diet … although when I was pregnant I didn’t. […] I’m not a person that takes pills. I don’t really want to start at this time when I’m supposed to be the most kind of wholesome" (Interview 19, age 37, prepared group).

“…this wasn’t a particularly planned pregnancy, so there was nothing… I wasn’t kind of priming my body with all of these clever vitamins and so on. So I was just trying to eat and be pretty healthy, but not related to pregnancy particularly” (Interview 13, age 39, absent pre-pregnancy period group).

“But I can barely…to be honest with you, I can barely remember to take the pill, let alone a vitamin. So for me if there was information out there that says you should take vitamins and stuff like that before you get pregnant you know, I think ‘Is this a gimmick from the pharmaceutical companies?’ […] Like again, like how do you know you should be taking vitamins? I don’t know. […] You don’t have studies to tell me that if I take [brand name of multivitamin product for pregnancy] all the time that my child’s going to come out a lot more healthier, not underweight, she’s going to be great. If you have studies on that, you know, I should be taking that throughout my whole pregnancy, then I would do it. But you’ve got to understand that I’m in a lucky position where I could afford to pay that … I don’t know, £15 a box every month, that’s fine … but how many women will actually go out and spend that amount of money on a precaution?" (Interview 20, age 30, poor knowledge group).

One woman from the prepared group, who was a supplement user outside of pregnancy, reported how her GP (outside of pregnancy) was dismissive of her supplement use: “My doctor always used to say to me ‘Be careful you don’t have expensive wee’” (Interview 7, aged 36, prepared group).

It is also worth noting that one woman (from the absent pre-pregnancy period group) who expressed anti-supplement views had a contrasting view about fortification of food with folate. Attitudes to fortification were not explored in the other interviews.

### Why did women invest in pre-pregnancy health and care?

The overall aim of the interviews was to find out why women invested in pre-pregnancy health and care, and with this in mind interviewers probed where possible throughout the interviews. In response, women who had carried out pre-pregnancy health and care actions (the prepared group) generally described themselves as trying to set the scene for positive outcomes. Their comments also conveyed a broad trust in available health information, e.g.:

Interviewer: “What made you decide to follow that information?”

R: “Um … I think it’s because … obviously there’s a proven track record, especially with the folic acid. So … and I’m the type of person that I wanted to do the right thing for my child and I was looking to have a child and I thought okay, let’s have a look and let’s see what I need to start doing now, you know in terms of diet change and stopping drinking and you know taking any vitamins or anything, so I started having a look around, and wanted to do the right thing for the baby.”

Interviewer: “Okay, was there any advice that you saw that you didn’t follow?”

R: “Um … let me think … no I didn’t really see … I’m just trying to think back. No not really. I mean … no I mean we were really keen to get pregnant, so that’s why I was trying to do everything I possibly could to make sure I was healthy. So no, there wasn’t anything that I thought ‘No I’m not going to follow this’ " (Interview 11, age 36, prepared group).

Interviewer: “Right. So when your nutritionist told you about them did you ever consider not taking them?”

R: “No, again I was keen to do the best thing I could do for the potential foetus. And also ‘cause of my age, you know thinking my body’s not going to be working as effectively as it would be if I was 20 years younger, so I want to maximise you know opportunities for a positive outcome.”

Interviewer: “Is there any advice or guidance you were given beforehand that you also followed or maybe didn’t follow?”

R: “Um … do you mean about supplements, that sort of thing?”

Interviewer: “Either supplements or general health or behaviours?”

R: “Yeah well I mean I didn’t drink, well hardly ever drank before, but I didn’t even want the taste of alcohol before with my first pregnancy, and certainly during the first pregnancy alcohol and coffee. So I hadn’t gone back onto them, ‘cause those were two things that might be problematic. But um … I mean as I say the advice that I’m aware of didn’t apply to me because I had no taste for them anyway. So easy” (Interview 17, age 40, prepared group).

In contrast, the comments of the poor knowledge and absent pre-pregnancy groups (i.e. that pre-pregnancy health and care actions were something that they did not know about or did not have the opportunity to carry out) ostensibly answered the ‘why’ question. There were, however, through the course of the interviews some hints to answer the further ‘why not’ question, ranging from not wishing to seek information to more active resistance or scepticism about pre-pregnancy health and care advice, e.g.:

Interviewer: “Can I ask generally … can you recall, or what do you know about any advice that’s given for women to choose about things to do before you become pregnant. What are you aware of?”

R: “Nothing. To be totally honest, nothing I think. I think I just relied on information from my family [about getting pregnant] to be honest with you. I don’t think … there is information out there but I just don’t think I was one of those people to go out and kind of look for it” (Interview 20, age 30, poor knowledge group).

Interviewer: “So yeah because you yourself were preparing and planning for pregnancy, it’s quite interesting to see where you were looking for that information, the kind of things that you were doing. Can you think of anything that, say, you found that you decided not to do?”

R: “In the run up to getting pregnant or in the dur- …?”

Interviewer: “No, prior to pregnancy.”

R: “No I can’t recall there was anything like … oh … yeah I don’t know, sorry I can’t recall there was anything that I decided not to do. I suppose I didn’t stop drinking, I guess I knew that I should probably in the best case scenario was to stop drinking, but I’m quite sociable and that was … I felt it was a big part of my lifestyle. And also I figured that like I would definitely stop drinking through the pregnancy, so … I wasn’t aware that it was as important if you know what I mean. I’m still not, to be honest, I don’t know that it’s as important to stop drinking before as it is during” (Interview 9, age 29, poor knowledge group).

“I remember my mum used to say that people change their mind between folic acid whether you do or you don’t, you know, things change over the years” (Interview 1, age 30 poor knowledge group).

Only one woman (from the absent pre-pregnancy period group), when discussing a pregnancy previous to her most recent one, described an actual disadvantage of carrying out a pre-pregnancy activity:

“I remember talking to my friend about whether she took folic acid while she was … you know when she started trying to get pregnant, and she said … I remember thinking is it going to make me more anxious [about getting pregnant] to be taking the folic acid” (Interview 4, age 34, absent pre-pregnancy period).

## Discussion

In our study there appeared to us to be three distinct groups in relation to pre-pregnancy health and care (the “prepared”, “poor knowledge”, and “absent pre-pregnancy period” groups), with levels of pregnancy planning and general attitudes to micronutrient supplementation being features in the formation of the groups. Broadly, the “prepared” group corresponded to the “high investors” recruited from the survey, and the “poor knowledge” and “absent pre-pregnancy period” groups with the “low investors”. The “prepared” group, who carried out pre-pregnancy activities such as taking folic acid and making changes to diet and lifestyle, had high levels of pregnancy planning and mostly positive attitudes to micronutrient supplementation outside of pregnancy. The “poor knowledge” group, who also had high levels of pregnancy planning, did not carry out pre-pregnancy activities such as taking folic acid and making changes to diet and lifestyle, although often had carried out other activities such as seeing a health professional for medication review. Women in this group described themselves as having no knowledge of folic acid supplementation or lifestyle recommendations, yet elsewhere in their interviews expressed a strong dislike of micronutrient supplementation with suspicion about its necessity. The last group, the “absent pre-pregnancy period” group, had the lowest levels of pregnancy planning and also expressed anti-supplement views. For this last group, discussing the pre-pregnancy period was difficult as response to questions quickly shifted to focus on pregnancy itself. For women in all three groups, the formal recognition of pregnancy usually led to (further) behaviour change.

### Limitations and strengths

The women in this sample tended to be older (there were few under age 25 and no teenagers) and well educated (two thirds had at least degree-level education). This socio-demographic composition reflected the original survey sample [24] and the slight bias towards older, well educated women among those who consented to be re-contacted. Due to the qualitative sampling criteria, the sample also had a high proportion of women who were not “White British” and who had a pre-existing health condition, however, neither of these criteria appeared to be determinants of women’s orientation to pre-pregnancy health and care. The sample was drawn from the ends of the distribution of investors in pre-pregnancy care, the high and the low investors. It is possible, therefore, that the “middling investors” (those with 2–5 pre-pregnancy activities) might have had different experiences and have expressed different views. The study was carried out in London (UK), a city which notably has extremes of wealth and a large black and ethnic minority population and therefore is different to many other areas of the UK. However, some reassurance can be gleaned from the fact that this study has similarities to the findings of other UK studies [20, 31–33].

Five interviewers carried out the 20 interviews. An advantage of this is that any personal interests or potential biases of particular interviewers were most likely diffused. A disadvantage is that interesting findings that emerged may not have been pursued in further interviews because of the discontinuity of interviewing, for example there was one clear instance of this when attitudes to the fortification of food with folic acid compared with supplementation in tablet form was not probed across interviews. Nine of the interviews were carried out face-to-face and 11 via telephone at the choice of the interviewee. The difference in the mode of interview may have influenced the content of the interviews, however we noticed no difference in length of interview or quality of content between the modes and most interviewers carried out both telephone and face-to-face interviews.

Probably the most important feature of the study in terms of the resulting findings is that we were clearly a publically-funded, health-related research team, with “Institute for Women’s Health, UCL”, “Department of Health Policy Research Programme”, and “NHS Research Ethics Committee” featuring in the participant information sheets given to women at recruitment. During the interviews, women will have made judgements about the nature of the study and the views held by the study team. Good interview technique can encourage interviewees to talk freely but the context of the study and its funding will still have framed these conversations. Where this is likely to be have been most important was in the conversations with women who did not carry out pre-pregnancy health and care activities (the poor knowledge and absent pre-pregnancy period groups). Their primary responses (i.e. that they did not know anything about pre-pregnancy health and care or were not in a position to do anything) can be seen as socially acceptable responses given to a health research team, effective in closing down further probing on a topic which they might hold, or at least assume they hold, different views to the interviewer. In a different study/interview context these interviewees might have framed their answers quite differently. Therefore, it is necessary for us to look at the entirety of their interviews and consider the influences that might have affected what they felt able to report.

### Comparisons with other studies

The fact that general attitudes to micronutrient supplementation were of key importance in our results was an unanticipated finding. Only one previous study, in the US, has found general attitudes to micronutrient supplementation to be important in relation to pre-pregnancy health and care [34]. Lindsey et al. examined attitudes to multivitamin use in relation to pregnancy among non-pregnant women. They found that non-users of multivitamins had distinctly different characteristics and attitudes, and self-reported more chaotic lifestyles. There was also a belief among this group that supplementation was not necessary, and that nutrients could be attained from normal balanced diet alone (even though most admitted they did not eat well). Further, non-users expressed an “all-or-nothing” position, in that unless they were carrying out other health behaviours (e.g. good diet, exercise, etc.) then there was no point in a multivitamin. In the UK, in some qualitative work evaluating the “Healthy Start” scheme (a scheme which provides low-income families with food vouchers and vitamin coupons for pregnant women, women in the year after pregnancy, and for children aged 6 months to 4 years), researchers found anti-supplement views among some parents, usually with parents believing that vitamin supplements were unnecessary if they and their children had a healthy diet, having a dislike of taking tablets and a concern that the vitamins might cause ill-health in children, plus citing the evidence of good health outcomes in previous pregnancies where no vitamin supplements had been used [33]. Outside of pregnancy, there have been studies comparing the lifestyles of supplement users with non-users and it is clear that supplement users generally have more nutritious diets and healthier lifestyles [35–40]. Dickinson and MacKay concluded, “Overall, the evidence suggests that users of dietary supplements are seeking wellness and are consciously adopting a variety of lifestyle habits that they consider to contribute to health living” [39]. Attitudes to micronutrient supplementation might be a shorthand way of identifying women’s potential orientation to pre-pregnancy health and care and is certainly worthy of future investigation in relation to pre-pregnancy care interventions.

Knowledge about the purpose of folic acid was vague among most interviewees in our study and awareness of the need for folic acid to be taken prior to pregnancy was poor, even among those who did take it before pregnancy. Poor knowledge relating to folic acid has been found in other qualitative studies in the UK [31, 32, 41, 42] and elsewhere [34, 43]. Goldberg et al., in a US study, found that women who knew that folic acid supplementation prevented birth defects were more than twice as likely to have taken folic acid before conception, and women who recognised that the timing of supplementation was important were five time likely to have taken folic acid before pregnancy [14]. In a review of folic acid public health campaigns, Rofail et al. reported that awareness, knowledge and consumption of folic acid improved following public health campaigns but that most women “still misunderstood the appropriate time to self-administer folic acid” [44]. Our findings suggest that despite the promotion of folic acid before pregnancy in the UK for two decades, the linking in women’s perceptions of folic acid with the pre-pregnancy period is still almost entirely absent.

In our study many women believed that they could obtain the nutrients they needed for conception and pregnancy from diet alone. A few studies have assessed this assertion empirically. In a study in Northern Ireland, McNulty et al. assessed the red cell folate (considered a reliable biomarker of the previous three months) of 296 women with singleton uncomplicated pregnancies at 14 weeks gestation [15]. They found that red cell folate levels were positively correlated with the reported duration of folic acid supplementation and were significantly lower in women who started folic acid after conception. They concluded that red cell folate concentrations in women not supplementing with folic acid supplementation were suboptimal in relation to the risk for neural tube defects. In a Dutch study, the diets of women planning pregnancy were assessed in detail using food diaries. The researchers found that most women’s diets exceeded the upper limit for saturated fat intake and the majority of women’s diets were sub-optimal in relation to copper, iron, selenium, and about half were deficient in vitamin A, with smaller proportions deficient in various B vitamins, vitamin C and vitamin E [45]. They also asked women about their own assessment of their diet and concluded that most women overrated the adequacy of their diets.

A number of women in our study, often those with pre-existing health conditions, spoke to health professionals about getting pregnant prior to pregnancy. It seemed as though the health professionals did not capitalise on this opportunity to deliver pre-pregnancy care, often not even mentioning folic acid to women. This rather lacklustre response from health professionals is consistent with our findings from interviews with health professionals (reported elsewhere [24]), which showed that health professionals’ knowledge was patchy, they wished for clearer guidelines to bring uniformity to their practice, and they were often not clear if pre-pregnancy care was their responsibility. It is interesting that one interviewee in our sample cited her GP’s comment about multivitamin supplements leading to “expensive wee”. This phrase stems from a Time magazine article in 1992 when Victor Herbert, a Professor of Medicine at New York City’s Mount Sinai medical school was quoted as saying, “We get all the vitamins we need in our diets. Taking supplements just gives you expensive urine.” [46]. In an article which discussed the “for” and “against” position for vitamin supplementation, it is ironic that this quote (part of the “against” position) should still resonate with health professionals in the UK over twenty years later. Hence, the attitudes of health professionals to micronutrient supplementation may also be important to the success of pre-pregnancy care. In a US survey of health professionals, investigating their knowledge and practice around the recommendation of folic acid supplementation, researchers found that the strongest predictor of a health professional recommending the use of folic acid supplements to patients was whether the provider took a multivitamin themselves [47]. Similarly, in a study of Australian GPs, where pre-pregnancy care was low on the list of health professionals’ priorities, they found that some GPs were also of the opinion that folate was not 100 % effective in preventing neural tube defects, and that even if they did discuss the use of folic acid their efforts might not be worthwhile because neural tube defects were still a possibility [48].

Women in the study carried out, and knew about, a fairly limited range of pre-pregnancy activities, most commonly taking folic acid, avoiding alcohol, stopping smoking, and healthy eating. Multiple micronutrient supplementation was assumed by many women to be necessary in pregnancy, and in pre-pregnancy by most of the “prepared” group, with women very familiar with commercial brands of multiple micronutrient products. Internationally, guidelines on pre-pregnancy care are heterogeneous [49] with the exception of uniformly recommending folic acid supplementation [50–53]. Several guidelines recommend the use of a multivitamin supplement as one component of an approach to ensure dietary sufficiency (other components include, for instance, achieving a healthy weight) [50–52]. Notably, guidance on pre-pregnancy care in the UK does not recommend the use of a multivitamin supplement, and does not otherwise discuss dietary sufficiency or supplementation (apart from recommendations to avoid taking high doses of Vitamin A, over-the-counter medicines and herbal remedies, and avoiding being obese) [53]. In contrast, UK National Health Service guidance does discuss dietary sufficiency and supplementation *in pregnancy* [54]. The consistency of advice and the linking from pre-pregnancy to pregnancy (a period that overlaps in practice given the time for pregnancy recognition to occur) could potentially be improved in the UK, particularly in light of scepticism about supplements (see earlier), evidence that micronutrient supplementation in early pregnancy can improve infant outcomes [55–58], and the dominance of the commercial sector in the UK in providing and promoting pregnancy and pre-pregnancy supplements. One of the tensions regarding policy on pre-pregnancy multiple micronutrient supplementation is the lack of direct evidence that supplementation (apart from folic acid) during this period leads to positive pregnancy and child outcomes. Further research in this area could clarify optimal pre-pregnancy recommendations. Certainly much greater promotion of the wider range of pre-pregnancy health and care activities is needed if the number and range of pre-pregnancy activities carried out by women is to be increased.

In our study, when asked, women suggested various ways of promoting pre-pregnancy health and care however they tended to comment that they would probably ignore such information if they were not thinking of getting pregnant. A high proportion of women do plan their pregnancies and potentially would be receptive to pre-pregnancy information from proactive health professionals. There are further women who have positive, albeit less explicit, intentions/feelings towards pregnancy who become pregnant [24, 59, 60]; little is known about how this group would receive pre-pregnancy information or how they best should be approached. One of the challenges for the delivery of future pre-pregnancy care is how to encourage women at different stages of pregnancy planning/intention and reproductive lifecourse to consider it relevant to themselves. Clearly, the US Centers for Disease Control and Prevention are currently attempting to broaden its relevance by framing pre-pregnancy health as relevant to the whole reproductive lifecourse, e.g.:

*“Preconception health* refers to the health of women and men during their reproductive years, which are the years they can have a child. It focuses on taking steps now to protect the health of a baby they might have sometime in the future. However, all women and men can benefit from preconception health, whether or not they plan to have a baby one day. This is because part of preconception health is about people getting and staying healthy overall, throughout their lives. In addition, no one expects an unplanned pregnancy. But it happens often.” [61]

In our study, however, there was a group for whom a pre-pregnancy period of preparation simply does not exist (even though there may have been positive feelings/intentions toward pregnancy by some in this group). One study of couples not planning pregnancy found that personal health promotion was more motivating than health promotion relating to pregnancy [62], and in a study of young women not planning pregnancy the promise of beauty-related outcomes was found to be of greatest interest [34], a sentiment also echoed by some of our interviewees.

## Conclusions

We identified three groups of women in relation to pre-pregnancy health and care: a “prepared” group, a “poor knowledge” group and “absent pre-pregnancy period” group. Broadly, the “prepared” group corresponded to the “high investors” recruited into our study, and the “poor knowledge” and “absent pre-pregnancy period” groups with the “low investors”. Different pre-pregnancy health promotion and health care delivery strategies are likely to be needed for each of the groups. Among the “prepared group”, who were proactive and receptive to health care messages, greater availability of information and better response from professionals could improve the range of pre-pregnancy activities carried out. Among the “poor knowledge” group, a better response from health professionals might yield greater uptake of pre-pregnancy information. A different, general health strategy might be more appropriate for the “absent pre-pregnancy period” group. The fact that general attitudes to micronutrient supplementation were closely related to whether or not women carried out pre-pregnancy health and care activities warrants further investigation. Clarity on the recommendations relating to pre-pregnancy micronutrient supplementation in the UK (given that they are currently different to international guidelines) would probably be helpful to both health professionals and women. Finally, the relatively limited range of activities in women's perceptions of pre-pregnancy suggests there is yet considerable scope for broadening the range and content of pre-pregnancy care that is promoted.
